# Menstrual cup and risk of IUD expulsion – a systematic review

**DOI:** 10.1186/s40834-022-00203-x

**Published:** 2023-01-21

**Authors:** Nicola Bowman, Annette Thwaites

**Affiliations:** 1 University College London, London, UK; 2 Institute of Women’s Health, University College London, London, UK

**Keywords:** Intrauterine device expulsion, Menstrual cup, Menstrual hygiene product, Intrauterine device, Contraceptive agents

## Abstract

**Background:**

The menstrual cup is a safe, cost-effective, and environmentally friendly menstrual product which is increasing in usage, especially in younger women. The potential risk for concomitant menstrual cup use to increase IUD expulsion has been raised over the last 10 years, however, few studies assess this. This systematic review aims to identify, appraise and synthesize the current specific evidence on menstrual cup use and risk of partial or total IUD expulsion.

**Methods:**

PubMed, and the Cochrane Library were searched for publications available in English, until February 20th, 2021. Quantitative and qualitative studies, systematic reviews and case series reports were included. Websites of menstrual cup manufacturers LenaCup®, DivaCup®, Lunette®, AllMatters® and Saalt® were searched for warnings relevant to IUD expulsion.

**Results:**

Seven studies were included in this review, comprising 73 partial or total IUD expulsion events in patients with IUD contraception using menstrual cups. The case study reports included two individuals who each experienced two and three expulsions respectively. Of the seven publications, three reported expulsion rates of 3.7%, 17.3% and 18.6%. Time to expulsion ranged from less than one week to two and a half years. These three studies disagree on whether there is a statistically significant association between menstrual cup use and IUD expulsion.

**Conclusion:**

There is a possible association between menstrual cup use and increased risk of IUD expulsion and this information should be shared with patients. However evidence is scarce and high-quality randomised controlled trials are needed to address this risk and the impact of factors such as age, menstrual cup removal technique, pelvic anatomy, IUD type, and measures such as cutting the IUD strings short or delaying menstrual cup use for a period post-insertion. This research gap is limiting patients’ ability to make informed choices regarding intrauterine contraception and menstrual management and must urgently be addressed in the context of rising IUD and menstrual cup use, particularly among a younger demographic who are seeking highly effective contraception.

## Introduction

Menstrual cups are a safe method for menstrual hygiene, with no adverse effect on vaginal flora or infection rates compared with pads and tampons [1]. Menstrual cup use is increasing with the global market projected to grow during 2021-2025 [2]. Menstrual cups collect between 10 and 38 mL of blood and should be emptied every 4-12 hours. The rise in menstrual cup usage, particularly among younger people, may be in part due to their longevity, as menstrual cups can be reused for up to 10 years, appealing to environmentally conscious users [3]. Despite a greater initial cost than non-reusable menstrual products, menstrual cups overall are more cost effective [1]. The website of menstrual cup manufacturer Mooncup® includes a calculator for consumers to estimate the financial savings they can expect from purchasing a menstrual cup [4].

Intrauterine contraceptive devices (IUD) are the most commonly used form of Long-Acting Reversible Contraception (LARC) globally with an estimated 159 million women of reproductive age (15-49 years) worldwide use an IUD as their primary method of contraception [5]. IUDs are one of the most effective methods of contraception, with an efficacy greater than 99% per year [6]. There are two main IUD types - hormonal IUDs and non-hormonal copper IUD (Cu-IUD), this is the terminology recommended by the World Health Organisation [7]. Hormonal IUD users are often amenorrhoeic, whereas a common side effect of Cu-IUDs is heavy menstrual bleeding. IUDs need to be inserted into the uterus by a trained medical professional. Depending on specific IUD type and region, IUDs are licensed for between 3 and 10 years and fertility returns immediately upon removal of IUD. Cu-IUDs can also be used as a form of emergency contraception [8, 9]. Recent literature has shown hormonal IUDs to also be effective as emergency contraception, however, the Faculty of Sexual and Reproductive Healthcare (FSRH) have not yet recommended the use of hormonal IUDs as emergency contraception for clinical practice [10, 11].

One of the risks associated with IUDs is unintentional expulsion which is reported to occur in 6% of the general population [12]. Expulsion is most common during menstruation and within the first year of insertion, particularly within the first 3 months [12]. Possible symptoms of IUD expulsion include abdominal or pelvic pain, and vaginal bleeding. Consequences of expulsion includes uterine perforation, and pregnancy due to altered position in the uterine cavity [13]. It is reported that younger patients have an increased risk of IUD expulsion and if replacement contraception not initiated, are at greater risk of pregnancy [6]. IUD expulsion may be asymptomatic and remain unnoticed until the IUD has exited the uterine cavity or remain undetected [14]. IUD expulsion can be subdivided into complete and partial expulsion [13]. In partial expulsion, the IUD may become displaced or mispositioned inside the uterus which may result in abdominal pain, heavy menstrual bleeding, and discomfort. Partial expulsion is confirmed via ultrasound scan of the uterus or pelvic examination by a medical professional. If asymptomatic, partial IUD expulsion may remain unnoticed by the user and therefore results in an increased risk of pregnancy [13].

Current FSRH guidance on intrauterine contraception written in 2019 states that menstrual cup use is not associated with increased risk of IUD expulsion [15]. However, there is conflicting evidence on the association between intrauterine contraceptive device (IUD) expulsion and concomitant menstrual cup use. The theoretical risk of downward suction pressure applied to the IUD or accidentally pulling on the IUD strings during menstrual cup removal have been described as potential mechanisms [16]. This review aims to assess the potential association between the use of menstrual cups with IUD expulsion.

## Methods

PubMed, and the Cochrane Library were searched on February 20th 2021, for publications available in English, without retrospective limit. Quantitative and qualitative studies, reviews and case series were included. The following search strategy was used across both databases: (IUD OR Intrauterine OR coil OR IUS OR LARC) AND (menstrual OR cup OR mooncup) AND (expel* OR expulsion OR eject* OR remov*). The reference lists of all relevant studies found in our initial search criteria were also reviewed, and the websites and information leaflets produced by menstrual cup brands (Lena Cup®, DivaCup®, Lunette®, AllMatters® and Saalt®) to look for warnings relevant to IUD expulsion [17].

**Fig. 1:**
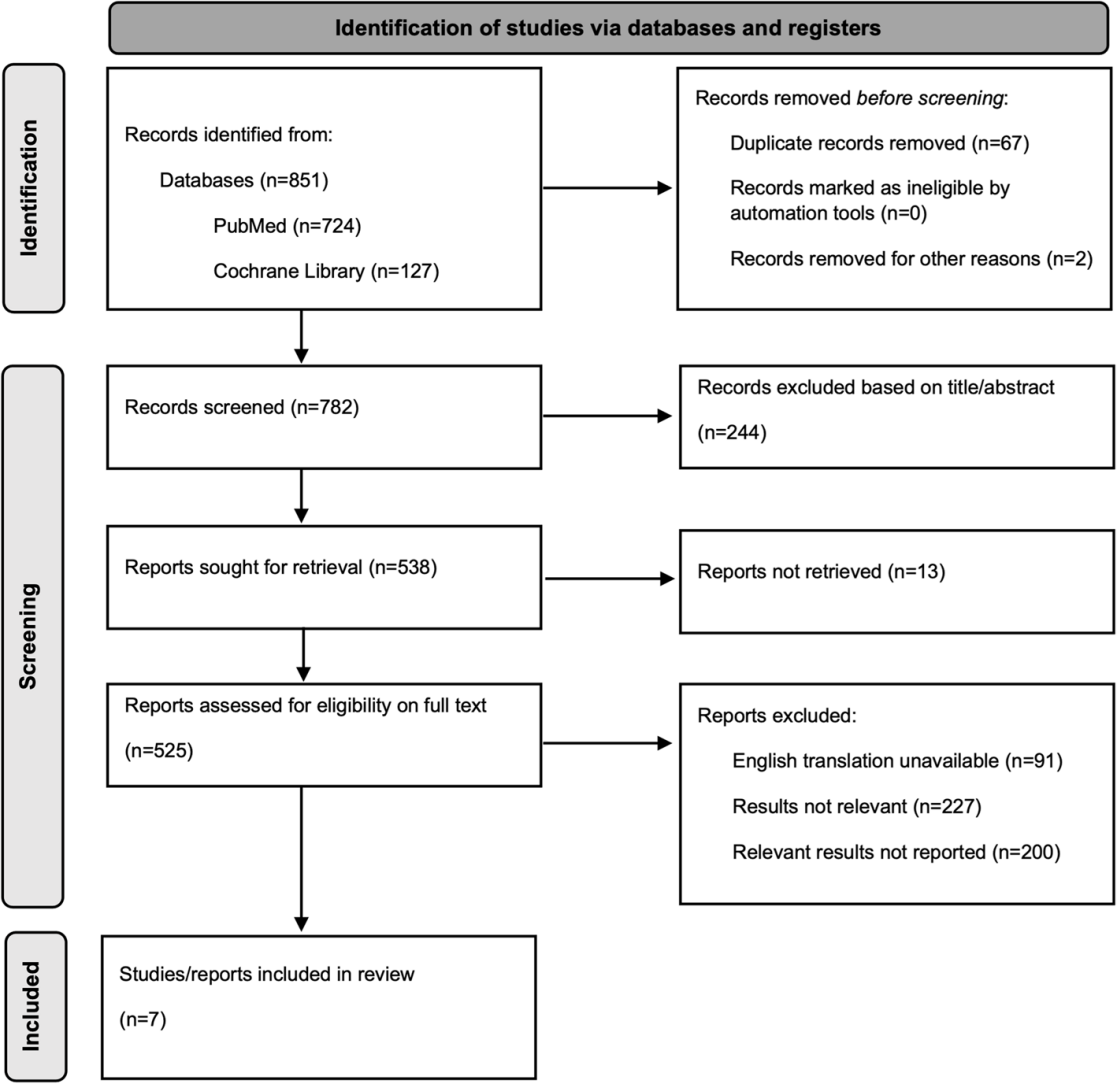
PRISMA Flow Diagram

Database search results were sorted using Zotero software. Duplicates were removed and an initial screening based on title and abstract was conducted by NB. To be eligible for inclusion, the study populations were concomitant users of menstrual cup and IUDs. Following initial screening, results underwent full text screening by NB and then reviewed for eligibility by AT. The identification and screening process is summarised in Fig. 1. Due to limited eligible studies, no publications were excluded based on study design, publication quality, or type of publication.

## Results

Initial database search strategy resulted in a total of 851 potentially eligible results; 724 from PubMed and 127 from Cochrane Library. Seven articles were included: two systematic reviews, one retrospective chart review, one internet based cross sectional survey, one abstract of an unpublished randomised control trial, and two articles regarding case reports. This topic was first explored in 2012 by the retrospective chart survey, then more recently by four further publications in 2019 and two publications in 2020. Characteristics of included studies and study conclusions are shown in Table 1. Publication characteristics are shown in Table 2.

**Table 1:**
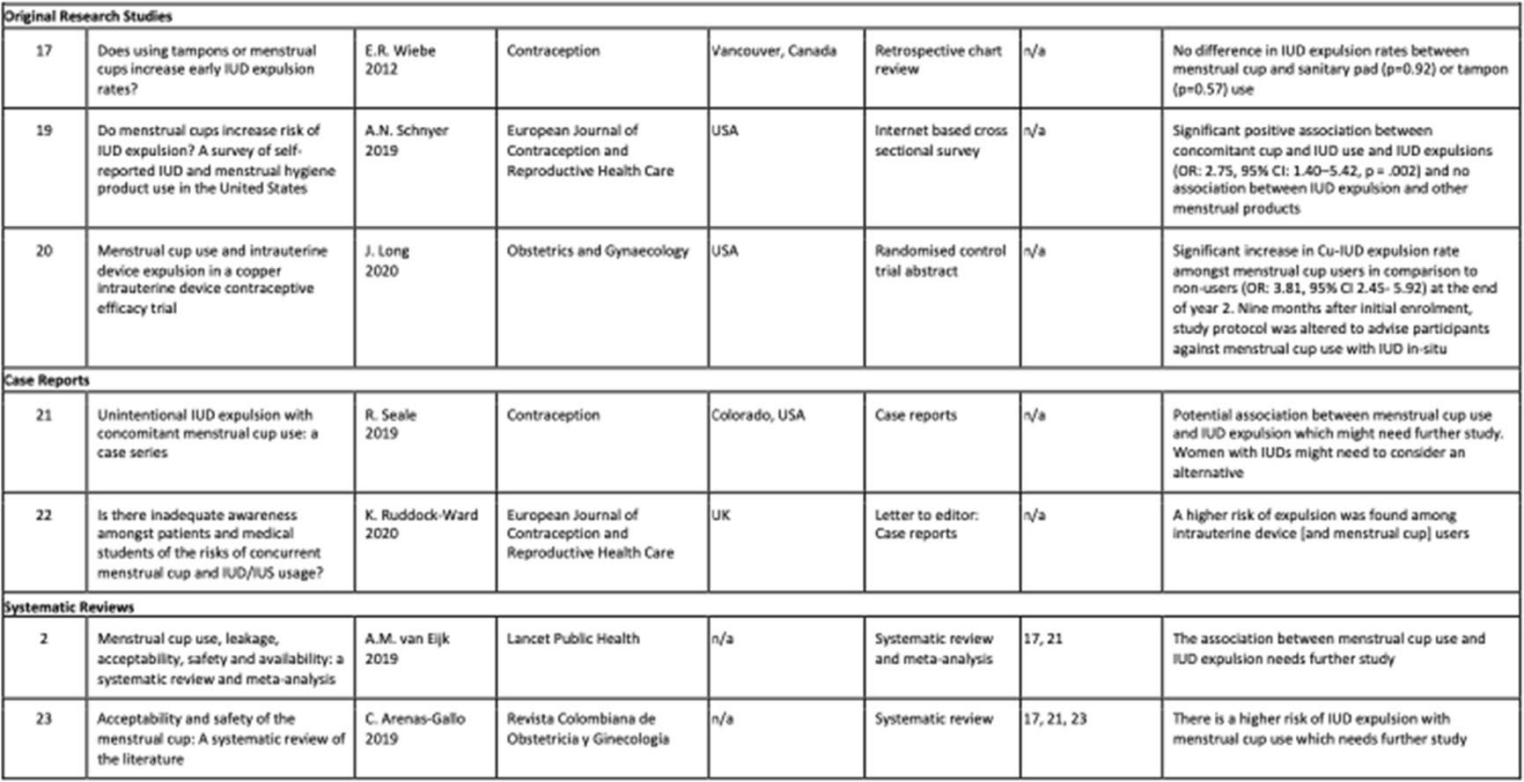
Characteristics of included studies

**Table 2:**
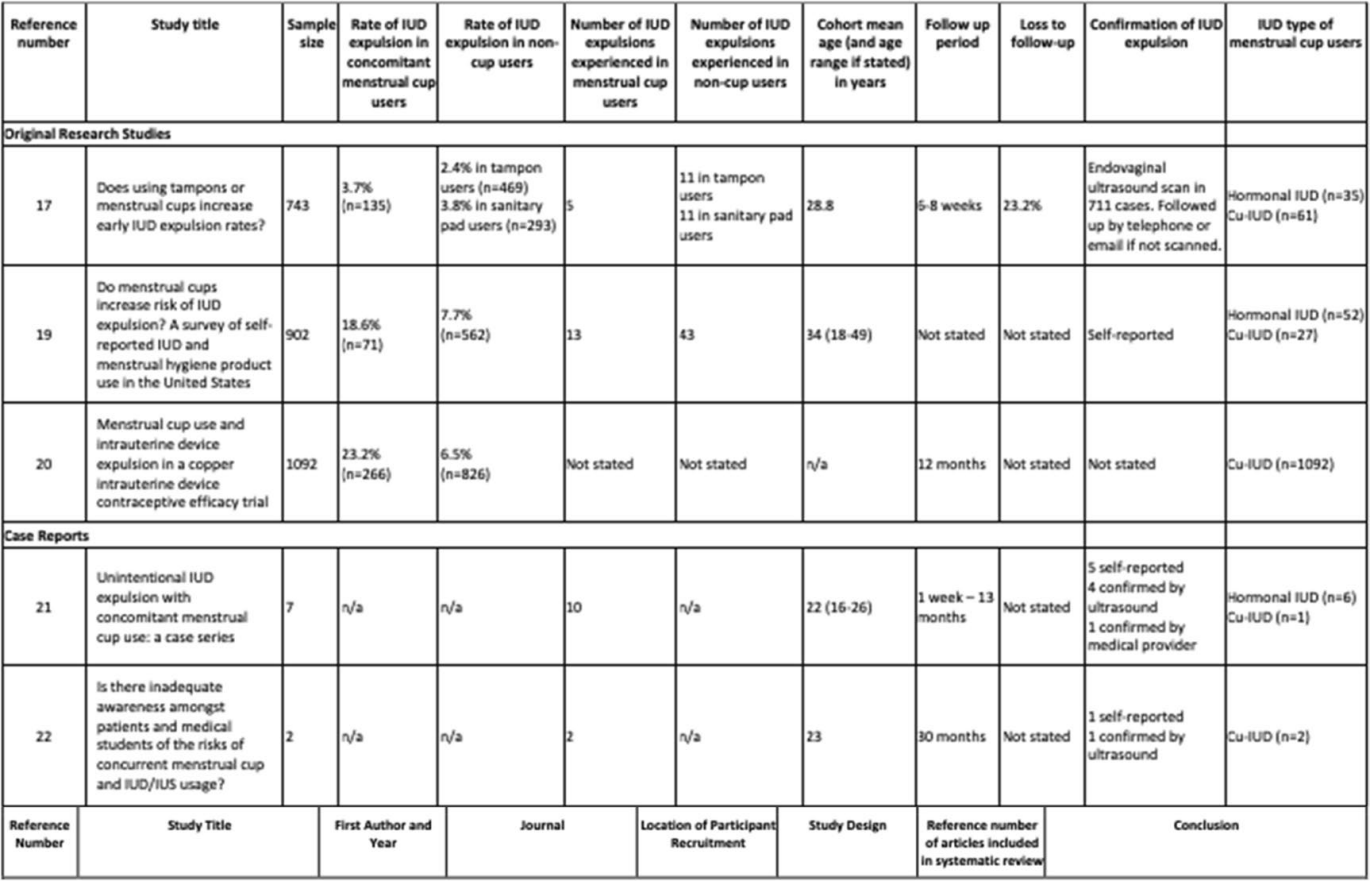
Rate of expulsion and cohort characteristics

Three studies provided rates of expulsion. Wiebe et al. concluded no significant association between concomitant menstrual cup and IUD use and increased risk of IUD expulsion [16]. Schnyer et al. concluded significant positive association between concomitant menstrual cup and IUD use and IUD expulsion [18]. Long et al. also concludes significant positive association, and 9 months after initial enrolment, the study protocol was altered to advise participants against use of menstrual cups with IUD in-situ due to the increased risk of expulsion [19].

Wiebe et al. assessed expulsion with endovaginal ultrasound at 6-week follow up [16]. Long et al. conducted follow up during the first-year post insertion at 6 weeks, and 3, 6, and 12 months, however specific data regarding time to expulsion is not available [19]. 74.5% of Schnyer et al. cohort reported expulsion occurring within the first year following IUD expulsion [18]. Time to expulsion ranged from under 1 week to 13 months in 7 cases reported by Seale et al., and 8 months up to 30 months in two cases reported by Ruddock-Ward et al. [20, 21].

The 2019 Lancet systematic review and meta-analysis assessed 3319 women for menstrual cup use, leakage, acceptability, safety, and availability, and concluded that menstrual cups are safe and effective [1]. This review included an additional FDA database case report of pain following menstrual cup removal but no observed IUD displacement on ultrasound but presented 2 months later with an ectopic pregnancy [1]. The review briefly mentions risk of IUD expulsion with concomitant use of a menstrual cup and suggests this may need further study as this risk cannot be excluded. Arenas-Gallo et al. reviewed acceptability and safety of the menstrual cup and concluded that there is an increased risk of accidental IUD removal associated with menstrual cup use [22].

## Discussion

The literature on menstrual cup use and IUD expulsion risk is scarce and limited. It includes lower levels of evidence such as case reports, an incompletely described abstract only randomised control trial, small studies with short follow up periods and subsections of larger reviews with a wider focus. The included studies were highly heterogenous in terms of methodology, type of IUD and menstrual cup, follow-up period, and assessment of IUD expulsion.

The ﻿single randomised control trial of over 200 concomitant IUD and menstrual cup users with a minimum of 12 months follow up is limited to the abstract only [19]. This randomised control trial reports a significant association between menstrual cup use and IUD expulsion but is missing key information on cohort characteristics, time to expulsion, menstrual cup type, removal method and pelvic anatomy. Additionally, this trial was conducted with Cu-IUD users, therefore the effect of menstrual cup use on hormonal IUD expulsion risk was not considered. This abstract was published in April 2020 and as of October 2022, the results of this trial are yet unpublished.

Schnyer et al. had a larger sample of menstrual cup users despite not specifically recruiting menstrual cup users [18]. This may reflect a trend of increasing use of menstrual cups amongst Caucasian, younger, and highly educated individuals, as these characteristics were highly represented in survey responses [16, 18]. As this survey relied on self-reporting, answers could not be verified and may be impacted by recall bias.

Time to expulsion in the reported case studies and the Schnyer et al. cohort suggests a longer follow-up period is necessary to accurately assess correlation between menstrual cup use and IUD expulsion [18, 20, 21]. Long et al. had a follow up period of minimum 12 months and reported increased risk of Cu-IUD expulsion with concomitant menstrual cup use [19]. Wiebe et al. conducted follow up at 6 weeks post-insertion and did not assess the number of times participants had used a menstrual cup or an alternative menstrual product since IUD insertion [16]. Wiebe et al. reported no significant difference in rates of IUD expulsion between menstrual cup users and users of other menstrual products. This may be accounted for by the shorter follow up period of only 6 weeks and the smaller sample size compared to Long et al. [16, 19]. No published randomised control trials or cohort studies have followed patients up to 1-year post-insertion.

Cohort characteristics varied across studies and case reports. Due to limited numbers of concomitant IUD and menstrual cup users, study populations are small, heterogenous and incompletely described, so it is not possible to adjust for possible demographic confounding factors such as BMI, parity, and age, which affects likelihood of IUD expulsion [23, 24]. Wiebe et al. results showed that menstrual cup users are more likely to be Caucasian, younger, and educated, however similar data was not included in the other studies in this review [16]. The greater age range represented in Wiebe et al. and Schnyer et al. studies may not accurately represent the younger patient cohort who is most at-risk of expulsion due to concomitant IUD and menstrual cup use [6, 16, 18]. The younger cohort included in the case series may more accurately reflect demographics associated with concomitant menstrual cup and IUD use [20, 21].

Menstrual cups vary in size, shape and material between manufacturers which may impact ease of removal and likelihood of IUD expulsion. Only Seale et al. included data on menstrual cup brand therefore we are unable to make conclusions regarding varying IUD expulsion risk between different menstrual cup brands [20]. Menstrual cups may fit differently depending on individual pelvic anatomy, for example a low cervix, smaller uterine cavity, or malformation/distortion of uterine cavity, which may in turn affect the menstrual cup removal technique and/or likelihood of IUD expulsion [13]. The studies included in this review did not include ultrasound assessment of pelvic anatomy, therefore, do not provide data on whether pelvic anatomical variants influence rates of IUD expulsion with menstrual cup use.

However, there is evidence to suggest that participants who chose to have a Cu-IUD reinserted following previous IUD expulsion have a higher expulsion rate with subsequent IUD insertions [15, 25]. This may suggest an anatomical propensity for repeated Cu-IUD expulsion, which could be exacerbated by menstrual cup use. In clinical practice, assessment of pelvic anatomy at the time of IUD insertion is mainly limited to bimanual examination. Ultrasound assessment may be advisable for patients with repeat IUD expulsion events or known abnormal pelvic anatomy to provide advice regarding use of menstrual cups following IUD insertion.

All of the menstrual cup manufacturers websites that were reviewed included information on concomitant IUD and menstrual cup use. All five of the manufacturers suggest that using menstrual cup with an IUD in-situ is acceptable for most people but mention possible increased expulsion risk and encourage discussing any queries with a medical professional [26–30]. Three manufacturers reference Wiebe et al. [26, 29, 30], the single study that concluded there was no association. Saalt® references all three of the original research studies assessed in this review and clearly summarises the conclusions of each study for the consumer. Removal instructions provided by two manufacturers both advise gently pulling on the stem initially before releasing the seal [26, 27], whereas the three other manufacturers emphasise releasing the suction first by pinching the base of the cup [28–30]. The manufacturers all included variations of more thorough safety netting advice, such as checking location of IUD strings after each period [29, 30], delaying menstrual cup use following IUD insertion [26, 27, 29], and cutting IUD strings shorter [27, 29, 30]. Further randomised control trials to conclude whether these interventions are effective at reducing incidence of IUD expulsion will help patients make fully informed decisions regarding choice of menstrual product and promote consistency in manufacturers advice.

Seale et al. propose two possible mechanisms for IUD expulsion with menstrual cup use [20]. Firstly, the accidental pulling of IUD strings during cup removal by the patient or IUD movement because of the downward suction applied to IUD strings created during menstrual cup insertion and removal. It is also possible that the IUD strings may be caught mechanically during menstrual cup removal. As mentioned by Seale et al. patient counselling on correct menstrual cup removal and cutting IUD strings may help reduce this increased risk of expulsion. Further research to conclude whether these interventions are effective at reducing incidences of IUD expulsion could help patients make fully informed decisions concerning choice of menstrual product with IUD in-situ.

Hormonal IUDs are slightly more effective at preventing pregnancy than Cu-IUDs [31]. The different bleeding profiles of Cu-IUDs and hormonal IUDs may affect expulsion rates differently. A common side effect of Cu-IUDs is heavier menstrual bleeding. Hormonal IUDs are known to cause hypomenorrhea, therefore, hormonal IUD users are less likely to use menstrual cups. Both Wiebe et al. and Schnyer et al. report that menstrual cup users in their cohorts were more likely to have a Cu-IUD [16, 18]. However, given the small sample size of concomitant Cu-IUD and menstrual cup users subgroup analysis by IUD type is not possible.

Patients who choose IUD discontinuation may face barriers to removal including clinical objection, difficulty accessing contraception services, appointment waiting times, and cost [32]. There has been growing interest in intentional IUD self-removal to bypass barriers for removal. Analysis of online content has shown a growing interest in online resources offering guidance for intentional IUD self-removal [33]. IUD self-removal is safe and low risk; however success rates are estimated to be 20% [32, 34]. Given the lack of evidence regarding the risk of  menstrual cup use and IUD expulsion this should not be recommended as a mechanism to facilitate IUD self-removal, and therefore this topic requires further investigation.

Research is limited on the knowledge and practice of healthcare professionals regarding this potential association between menstrual cup use and IUD expulsion. Ruddock-Ward et al. note that neither patient was aware of any potential increased risk of IUD expulsion with menstrual cup use prior to the IUD expulsion event [21]. One patient decided against IUD replacement for fear of a repeated expulsion. Schnyer et al. reported most IUD users (69.8%) received ‘no specific guidance’ from their provider about period protection while using an IUD, and 8% were told to use only pads and/or tampons [18]. There is no UK guidance that suggests clinicians should advise regarding a potential association, yet some experienced clinicians do so based on their anecdotal evidence.

This review is the first literature search of which we are aware which is focussed specifically on the risk of IUD expulsion with concomitant use of menstrual cups and IUDs. However, the literature on menstrual cup and IUD expulsion risk is scarce and largely limited to lower levels of evidence (case studies or abstracts only). The screening process for this review was conducted by a single reviewer (NB). However, AT verified the included studies met the inclusion criteria and no subjective exclusion criteria were used. Large randomised control trials with adequate follow up periods are needed to address potential associated factors such as age, parity, cup manufacturers, IUD type, pelvic anatomy, menstrual cup insertion/removal techniques and whether expulsion risk can be mitigated by cutting the IUD strings short or delaying use of menstrual cup for a period after IUD insertion or expulsion.

## Conclusion

Since this theoretical risk of concomitant menstrual cup use and increased IUD expulsion was first explored in 2012, evidence is scarce and calls for high quality randomised control trials to determine this remain unanswered. A single small study in 2012 with a 6-week follow up period did not find significant association between concomitant IUD and menstrual cup use and increased risk of IUD expulsion, however, subsequent studies have supported an association. Manufacturers are not incentivised to promote this research or make reliable recommendations. This research gap is limiting patients’ ability to make informed choices regarding intrauterine contraception and menstrual management. This research gap must be urgently address in the context of rising IUD and menstrual cup use, particularly among a younger demographic who are seeking highly effective contraception.

## Data Availability

The data that support the findings of this review are openly available in the databases Pubmed and Cochrane library. PMID: 31324419 - 10.1016/S2468-2667(19)30111-2 PMID: 22464406 - 10.1016/j.contraception.2011.12.002 PMID: 31335218 - 10.1080/13625187.2019.1643836 PMID: 30981842- 10.1016/j.contraception.2019.03.047 PMID: 32852236 - 10.1080/13625187.2020.1807000 PMID: 32770872 - 10.18597/rcog.3425 Cochrane Library ID Number - CN-02214862.

## Abbreviations

IUD: Intrauterine Device
Cu-IUD: Copper Intrauterine Device
IUS: Intrauterine System
LARC: Long-Acting Reversible Contraception
LNG-IUD/LNG-IUS: Levonorgestrel-releasing Intrauterine Device/System
FDA: Food and Drug Administration
FSRH: Faculty of Sexual and Reproductive Healthcare
NB: Nicola Bowman (Author)
AT: Annette Thwaites (Author)
