# Correction: Menstrual cup and risk of IUD expulsion – a systematic review

**DOI:** 10.1186/s40834-023-00219-x

**Published:** 2023-02-27

**Authors:** Nicola Bowman, Annette Thwaites

**Affiliations:** 1 University College London, London, UK; 2 Institute of Women’s Health, University College London, London, UK

**Correction: Contracept Reprod Med 8, 15 (2023)**

**https://doi.org/10.1186/s40834-022-00203-x**

Following publication of the original article [1], the authors reported that the Tables 1 and 2 were mistakenly listed.

The correct Table 1 should read:

**Table 1:**
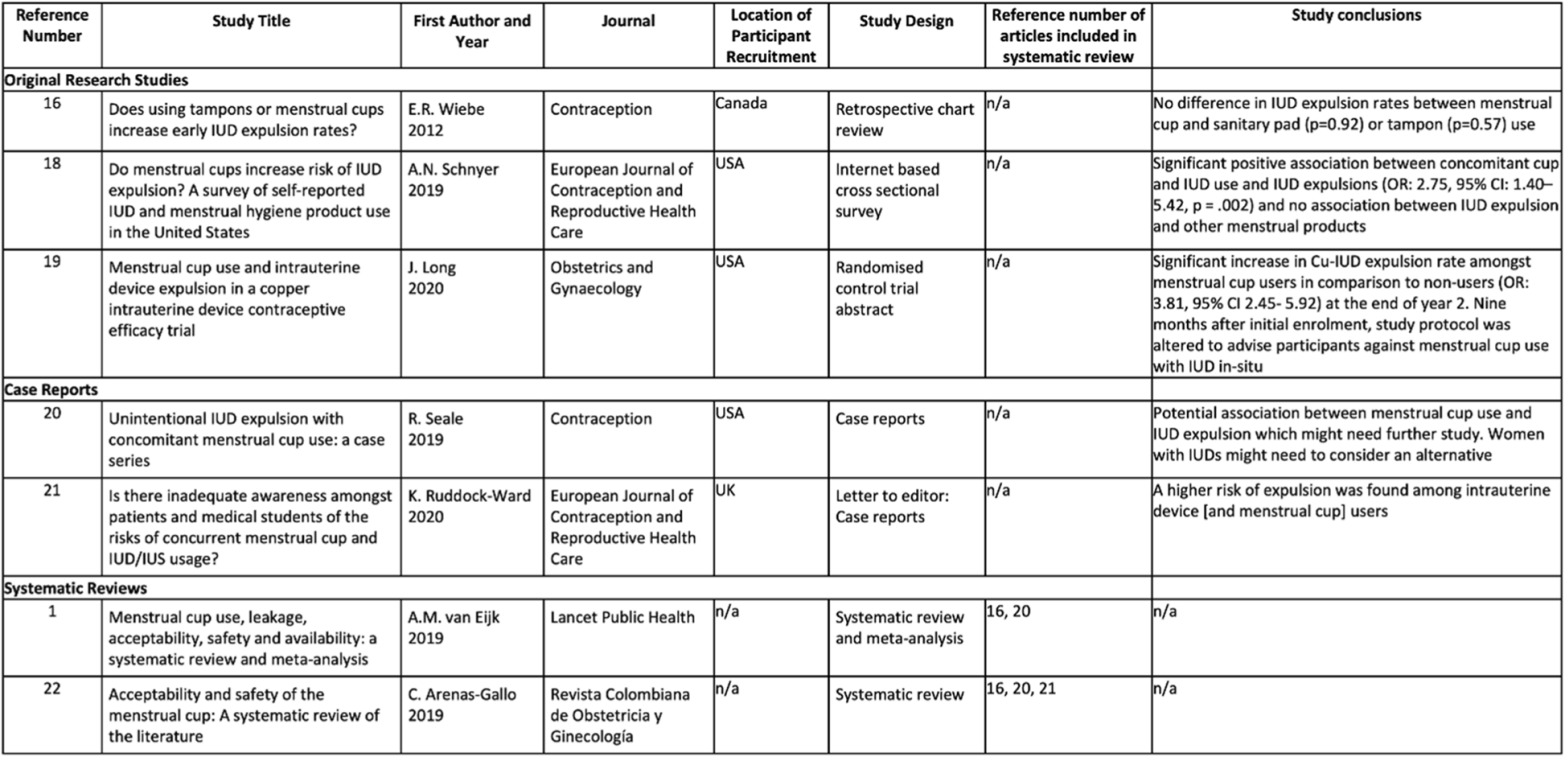
Characteristics of included studies

The correct Table 2 should read:

**Table 2:**
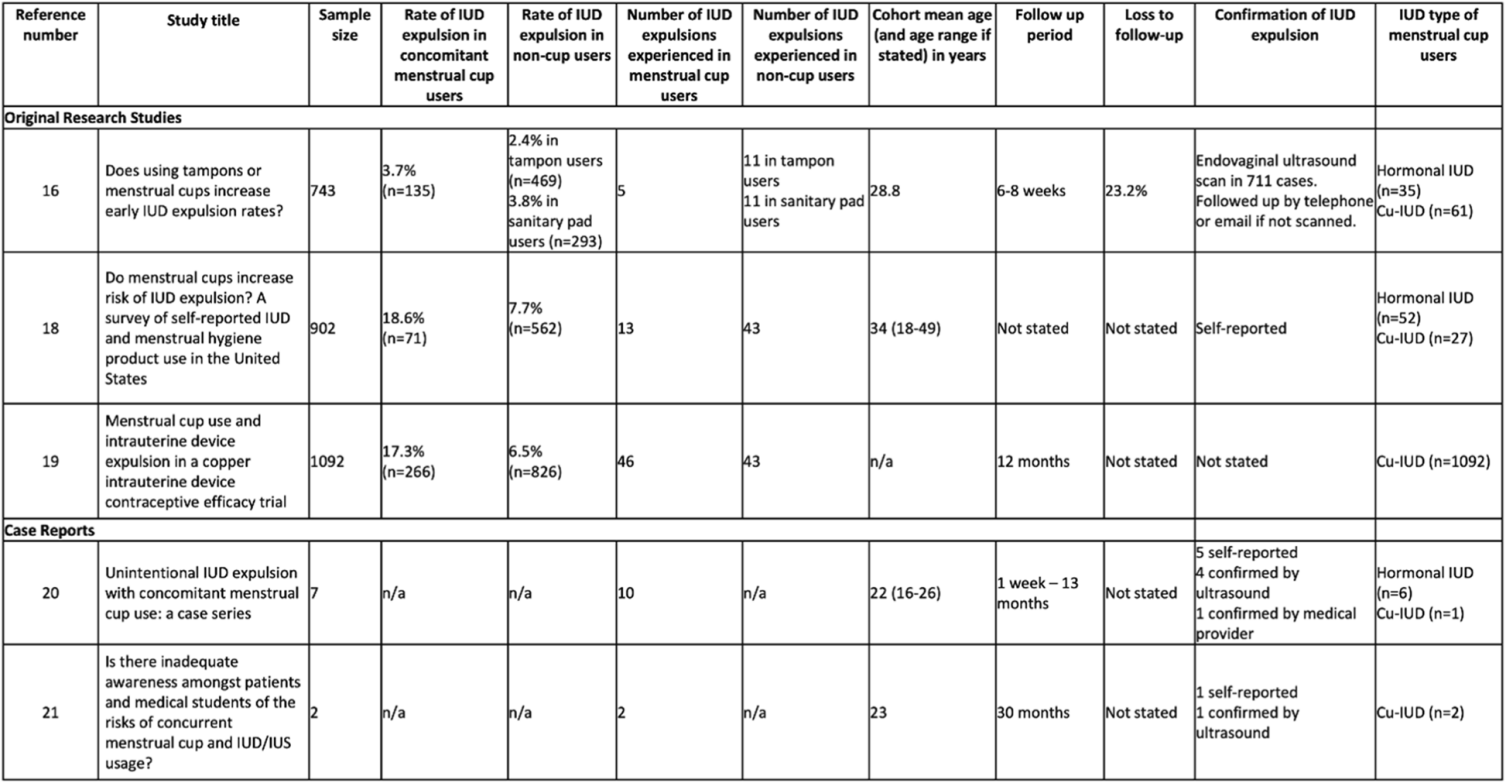
Rate of expulsion and cohort characteristics

The original article [1] has been updated.
